# Learning and the possibility of losing own money reduce overbidding: Delayed payment in experimental auctions

**DOI:** 10.1371/journal.pone.0213568

**Published:** 2019-05-23

**Authors:** Yu Yvette Zhang, Rodolfo M. Nayga, Dinah Pura T. Depositario

**Affiliations:** 1 Department of Agricultural Economics, Texas A&M University, College Station, Texas, United States of America; 2 Department of Agricultural Economics and Agribusiness, University of Arkansas, Fayetteville, Arkansas, United States of America; 3 Department of Agribusiness Management and Entrepreneurship, College of Economics and Management, University of the Philippines Los Baños, College, Laguna, Philippines; Universidad de Alicante, SPAIN

## Abstract

In this study, we designed a delayed payment mechanism in laboratory second price auctions (SPAs), under which subjects received a cash endowment two weeks after the experiment day and had to use their own money to pay the experimental losses (if any) on the experiment day. We compared the effect of delayed payment on overbidding in the induced value SPAs with the conventional “on-the-spot” payment mechanism where the subjects received an endowment on the experiment day, and the prepaid mechanism where the subjects received the endowment two weeks before the experiment day. Each auction was repeated for 20 rounds to provide sufficient learning opportunities to the bidders. Our results showed that bids converged to the corresponding values over auction rounds and overbidding was reduced by previous losses, consistently with the adaptive learning theory. Moreover, overbidding seems to depend significantly on bidders’ cash holding, and the magnitude of the payment treatment effects depends crucially on liquidity constraints. In the presence of liquidity constraints, both delayed and prepaid payment mechanisms reduced overbidding, while in the absence of liquidity constraints, only the delayed endowment mechanism reduced overbidding. Furthermore, when controlling the degree of liquidity constraints, subjects with higher GPAs were less likely to overbid and the delayed endowment mechanism significantly reduced overbidding compared to other payment mechanisms. These results suggest that overbidding in SPAs might be caused by bounded rationality and could be reduced by adaptive learning especially when overbidding becomes “truly” costly to subjects.

## 1. Introduction

It is well known that in second price sealed bid auctions (SPAs), bidding one’s true value is a weakly dominant strategy [1]. However, many laboratory experiments have reported significant and persistent overbidding in SPAs, i.e., bidding above one’s value [2, 3]. One explanation for overbidding is based on nonstandard preference such as utility from winning (“joy of winning”) [4, 5] or disutility from losing (“spite”) [6, 7]. Another explanation is “bounded rationality”, under which systematic errors in reasoning are hypothesized to cause overbidding. Subjects might have the misconception that overbidding improves the probability of winning with little cost because the winner only pays the second-highest bid [2]. Furthermore, in a laboratory setting, subjects could have difficulty learning not to overbid as overbidding rarely costs them money [3].

The objective of this study is to use a payment mechanism that would reduce overbidding in laboratory experiments. In auction experiments, it is a common practice to pay subjects an initial endowment or participation fee at the time of the experiment, as monetary incentive for them to behave as in real-life situations (“on-the-spot” payment mechanism). One disadvantage of “on-the-spot” payment is that, after receiving the payment people may become more risk-loving as they may not consider the easily-gotten income as their own money, which is referred to as the “house money effect” [8]. If bidders derive utility from winning beyond the monetary payoffs (“joy of winning”), their risk-loving behavior induced by the “house money effect” may cause overbidding [4, 5, 9, 10]. Another disadvantage of “on-the-spot” payment is that subjects do not receive a strong feedback for learning to correct the mistakes of overbidding (bounded rationality), because with the initial money endowment, the net gain of a subject is usually positive even when incurring a loss [3, 11]

One possibility of mitigating the overbidding problem is to have subjects use their own money in the experiments [12, 13]. This approach has two possible advantages. Firstly, subjects may become more risk-averse and are less attracted to winning in the absence of the house money effect. Consequently, overbidding will be reduced if it is driven by “joy of winning”. Secondly, subjects may learn effectively when they have to pay the cost of overbidding using their own money [14]. According to the adaptive learning theory, if overbidding represents a mistake in decision making, subjects may learn the optimal strategy based on prior observations in repeated games, without necessarily understanding the nature of the equilibrium [15, 16]. However, if participants are not paid and have to use their own money in the experiments, the method is not ethical and may lead to selection bias. Instead, a number of studies used a “prepaid mechanism,” under which the initial endowment was paid to subjects prior to the actual experiments [17, 18, 19]. Rosenboim and Shavit [19] found a higher degree of risk-aversion and lower bids in prepaid sessions compared with the “on-the-spot” payment session in SPAs.

What is novel in our study is that we designed a “Delayed Payment Mechanism”, inspired by the prepaid mechanism [17, 18, 19]. We conjecture that by paying the subjects two weeks after the experiments, the possible influence of windfall gains on subjects’ bidding behavior may be eliminated and overbidding could become “truly” costly to the subjects since subjects have to pay their experimental losses using their own money on the experiment day. The degree of overbidding was quantified by the induced-value auctions [20], in which subjects bid for a fictitious good with pre-assigned values and received payment equal to the difference between their assigned induced value and the winning price if they won the auctions. To test if overbidding is caused by bounded rationality and whether it can be reduced by adaptive learning, each auction was repeated for 20 rounds to provide subjects with sufficient learning experiences. Our results show that losses from previous rounds reduced overbidding, and the “delayed endowment” mechanism reduced overbidding significantly compared with the commonly used “on-the-spot” payment mechanism. Taking into account liquidity constraints, we found that both delayed and prepaid payments reduced overbidding among the subjects with liquidity constraints, especially in the last 10 auction rounds. However, in the absence of liquidity constraints, overbidding was reduced only under the delayed endowment mechanism but not under the other payment mechanisms. Furthermore, controlling the degree of liquidity constraints, we found that subjects with higher GPAs were less likely to overbid and the delayed endowment mechanism significantly reduced overbidding compared to other payment mechanisms. These findings suggest that overbidding in SPAs might be caused by bounded rationality and could be reduced by adaptive learning especially when overbidding becomes “truly” costly to subjects.

The rest of paper is organized as follows. We will first present the experimental procedures, which include the second-price auctions and elicitations of time and risk preferences. Then we will report and discuss the experiment results, and conclude the paper.

## 2. Experimental methodology and procedure

The experiments were conducted in the College of Economics and Management, University of Philippines Los Baños (UPLB). The Texas A&M University Institutional Review Board (IRB) approved this study. All participants have provided written informed consent to participate in this study and the IRB approved this consent procedure.

### 2.1 Payment schemes

One hundred and twenty undergraduate students were recruited and randomly assigned to four payment treatment sessions with 30 subjects in each session:

- **“on-the-spot” payment**: a lump sum payment (cash endowment plus/minus experimental earnings/losses) was paid to each subject at the end of the experiment;
- **lump sum delayed payment**: a lump sum payment (cash endowment plus/minus experimental earnings/losses) was delivered to each subject two weeks after the experiment;
- **delayed endowment**: cash endowment was delivered to each subject two weeks after the experiment. However, the subjects received the experimental earnings, or paid the experimental losses on the day of the experiment;
- **prepaid payment**: cash endowment was delivered to each subject two weeks prior to the experiment while the subjects received the experimental earnings, or paid the experimental losses on the day of experiment.

The cash endowment for each subject was 150 Philippine Pesos (PHP) (1USD = 41.59PHP), which included 100PHP as participation fee and 50PHP as starting balance for the auctions. To facilitate the delivery of the payment, we recorded the subjects’ class schedules, classrooms, and/or contact addresses when they signed up for the experiments. On the experiment day we provided each participant a promissory note promising the money delivery. The payment was then delivered to the subjects in their classrooms on the scheduled payment day. Note that when surveyed about whether they trusted us for receiving delayed payment, all subjects answered “Yes”.

### 2.2 Auction designs

In each treatment session, subjects were randomly assigned to three auction groups to participate in 10-bidder second price induced value auctions. They were instructed to bid for a fictitious good that had some ‘value’ to them and each auction was repeated for 20 rounds. In each auction around, every subject was assigned a private value for this good, and the person who submitted the highest bid among the 10 bidders was declared the winner of the group. If more than one bidder submitted the highest bid in a group, the winner was randomly selected among them by rolling a dice. The winner earned a profit equal to his or her assigned value minus the second highest bid. All other bidders received a zero profit. Note that the winner’s profit would be negative if the second highest bid is higher than the assigned value. Bidders’ values were randomly drawn from a uniform distribution on the interval [1,100] and were different for each bidder in every round. The values and bids were described to the subjects in the unit of Experimental Dollars (ED) but final payment to the subjects were converted from EDs to PHPs (10ED = 1PHP). Each individual had no information about the other subjects’ values. Following Lusk et al. [21], we used the same set of 200 values (10 bidders x 20 auction rounds) in all sessions.

At the end of each auction round, the subjects were informed about the profits they earned (or losses if the profit became negative) from that round and their accumulated profit (or losses) up to that round, which was the sum of profits from all the completed rounds. This information was made known to each subject privately. If at any round, a subject’s accumulated profit up to that round became less than -500ED, he/she was no longer allowed to bid in subsequent rounds as this meant that the subject had exhausted the starting balance of 50 PHP (10ED = 1PHP). Note that this situation only occurred once throughout all sessions.

### 2.3 Elicitation of risk and time preferences with varying background consumptions

In the real world, personal income and background consumptions may differ across individuals and vary over time. According to the prospect theory, outcomes are evaluated using a value function defined over departures from a reference point [22, 23], and subjects’ risk and time preferences can be affected by the temporal reference point [24, 25]. Our experiment provides a compelling case of non-constant background consumption and differential temporal reference points since subjects received the endowment on different days due to different payment schemes.

We modified the method of Andersen et al. [26] to elicit subjects’ time preference and risk preference jointly, allowing the possibility of varying background consumption. The multiple price list table of time preference survey is presented in Table 1 [27, 28]. Consider two payment scenarios: (i) receiving *M*_*t*_ at time *t* but no income at a later time period *t* + 1, (ii) receiving no income at time *t* but *M*_*t+τ*_ at time *t* + *τ*. Under the assumption of exponential time discounting, a subject was indifferent between these two income options if and only if,

$$U(w_{t}+M_{t})+\frac{U(w_{t+1})}{1+\delta }=U(w_{t})+\frac{U(w_{t+1}+M_{t+1})}{1+\delta },$$ (1)

where the utility function *U(x)* is separable and stationary over time, *δ* is the time discount rate, and *w*_*t*_ and *w*_*t*+1_ are the background consumption at time *t* and *t* + 1, respectively. Assuming CRRA utility function, *U*(*x*) = *x*^(1 − *r*)^/(1 − *r*), where *r* is the CRRA coefficient (*r* ≠ 1), solving Eq (1) yields the subject’s time discount rate,

$$\delta =\frac{{{(w}_{t+1}+M_{t+1})}^{1-r}-{{(w}_{t+1})}^{1-r}}{{\left(w_{t+}M_{t}\right)}^{1-r}-{{(w}_{t})}^{1-r}}-1.$$ (2)

**Table 1:** Payoff matrix of time preference survey.

Payoff Alternative	Payment Option A (Pays today)	Payment Option B (Pays in 2 weeks)	Preferred Payment Option Write A or B
1	PHP 50.00	PHP 52.00	
2	PHP 50.00	PHP 54.00	
3	PHP 50.00	PHP 56.00	
4	PHP 50.00	PHP 58.00	
5	PHP 50.00	PHP 60.00	
6	PHP 50.00	PHP 62.00	
7	PHP 50.00	PHP 64.00	
8	PHP 50.00	PHP 66.00	
9	PHP 50.00	PHP 68.00	
10	PHP 50.00	PHP 70.00	
11	PHP 50.00	PHP 72.00	
12	PHP 50.00	PHP 74.00	
13	PHP 50.00	PHP 76.00	
14	PHP 50.00	PHP 78.00	
15	PHP 50.00	PHP 80.00	
16	PHP 50.00	PHP 82.00	
17	PHP 50.00	PHP 84.00	
18	PHP 50.00	PHP 86.00	
19	PHP 50.00	PHP 88.00	
20	PHP 50.00	PHP 90.00	

We divided a subjects’ monthly allowance provided in the post-auction survey by 30 days and used that as his regular daily consumption *w*. For the “on-the-spot” payment treatment, the background consumption on the experiment day was his regular daily consumption plus experiment payment, whereas the background consumption two weeks after was the regular daily consumption. For the lump sum delayed payment treatment and the delayed endowment treatment, the background consumption on the experiment day was the regular daily consumption, while background consumption two weeks after was regular daily consumption plus experiment payment. For the prepaid treatment, the background consumptions on experiment day and on the day two weeks after were both the same as the regular background consumption, as a subject received the endowment two weeks before the experiment. The CRRA risk coefficient *r* was elicited following the method of Andersen et al. [26] and Holt and Laury [29] as shown in Table 2.

**Table 2:** Payoff matrix of the risk preference survey.

Payoff Alternative	Lotter A				Lottery B				Preferred Lottery Write A or B
	*p*	PHP	*p*	PHP	*p*	PHP	*p*	PHP	
1	0.1	50.00	0.9	40.00	0.1	96.25	0.9	2.50	
2	0.2	50.00	0.8	40.00	0.2	96.25	0.8	2.50	
3	0.3	50.00	0.7	40.00	0.3	96.25	0.7	2.50	
4	0.4	50.00	0.6	40.00	0.4	96.25	0.6	2.50	
5	0.5	50.00	0.5	40.00	0.5	96.25	0.5	2.50	
6	0.6	50.00	0.4	40.00	0.6	96.25	0.4	2.50	
7	0.7	50.00	0.3	40.00	0.7	96.25	0.3	2.50	
8	0.8	50.00	0.2	40.00	0.8	96.25	0.2	2.50	
9	0.9	50.00	0.1	40.00	0.9	96.25	0.1	2.50	
10	1	50.00	0	40.00	1	96.25	0	2.50	

The subjects were asked to choose between lottery A and lottery B in each row. Each lottery offers a probability *p* of receiving a high payment, and a probability *1-p* of receiving a low payment. Given *p* of the row where a subject switches from choosing A to choosing B, the CRRA coefficient *r* can be calculated, with *r>0* indicating risk aversion, *r<0* indicating risk loving, and *r = 0* indicating risk neutrality.

## 3. Results and discussion

### 3.1 Summarization of auction results

Descriptive statistics and definition of variables used in our analysis are presented in Table 3, and summary statistics of the bids are presented in Table 4. Consistent with previous studies, we found substantial overbidding in all four treatments, with 80% of the bids exceeding the associated values.

**Table 3:** Definition of variables and descriptive statistics.

Variable	Description	Value
Number of Observations	Subjects with on-the-spot payment	30
	Subjects with lump-sum delayed payment	30
	Subjects with delayed endowment	30
	Subjects with prepaid payment	30
	Total Number of Observations	120
Age		Mean = 19.2, SD = 1.13
Gender	1 = Subject is female	65%
	0 = Subject is male	35%
Pocket Money (PHP)	Money brought to the experiment	Mean = 770, SD = 969
Monthly Allowance	0 = Less than PHP 999	34
	1 = PHP 1,000-PHP 1,999	32
	2 = PHP 2,000-PHP 2,999	19
	3 = PHP 3,000-PHP 3,999	10
	4 = PHP 4,000-PHP 4,999	7
	5 = PHP 5,000-PHP 5,999	9
	6 = PHP 6,000-PHP 6,999	4
	7 = PHP 7,000-PHP 7,999	2
	8 = PHP 8,000-PHP 8,999	1
	9 = PHP 9,000-PHP 9,999	2
Work Status	0 = Not working	105
	1 = Part-time working	14
	2 = Full-time working	1
GPA (4.0 Scale)	Grade point average	Mean = 2.22, SD = 0.51

**Table 4:** Summary of bids.

	On-the-Spot Payment	Lump Sum Delayed Payment	Delayed Endowment	Prepaid
**Mean for all 20 rounds (EDs)**				
**Bids**	75.4 (51.1)	75.8 (54.2)	67.1 (49.7)	75.2 (50.1)
**Overbid (bid–value)**	24.8 (40.5)	24.9 (44.7)	16.4 (35.7)	24.4 (40.0)
**Winning price** **(the second highest bid)**	124.8 (34.0)	125.0 (39.8)	109.4 (44.6)	123.83 (42.9)
**Mean for the first 10 rounds (EDs)**				
**Bids**	67.0 (49.7)	70.4 (56.5)	67.0 (59.9)	69.2 (49.6)
**Overbid (bid–value)**	21.2 (37.9)	24.1 (45.6)	20.6 (45.9)	22.7 (39.6)
**Winning price** **(the second highest bid)**	119.0 (38.3)	124.7 (46.1)	118.8 (60.8)	119.7 (46.5)
**Mean for the last 10 rounds (EDs)**				
**Bids**	83.6 (51.3)	81.1 (51.3)	67.4 (36.7)	81.2 (51.2)
**Overbid (bid–value)**	28.4 (42.7)	25.8 (43.9)	12.1 (20.3)	26.2 (40.4)
**Winning price** **(the second highest bid)**	130.5 (28.6)	125.0 (33.1)	100.1 (12.6)	128.0 (39.4)

Note: Standard errors of the mean in parenthesis.

**Fig 1:**
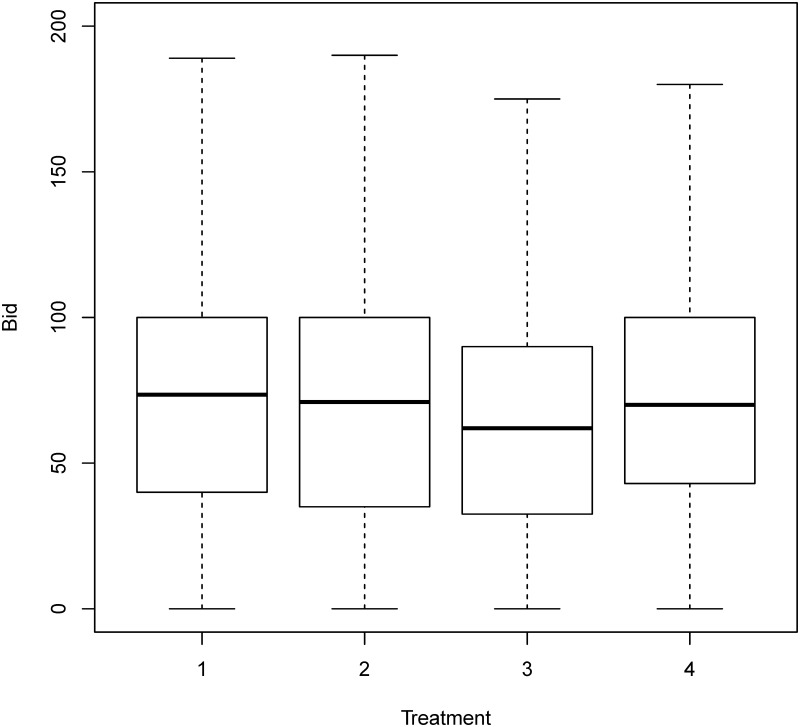
Bids in all four treatments. Treatment 1: On-the-spot payment; Treatment 2: Lump sum delayed payment; Treatment 3: Delayed endowment; Treatment 4: Prepaid payment.

**Fig 2:**
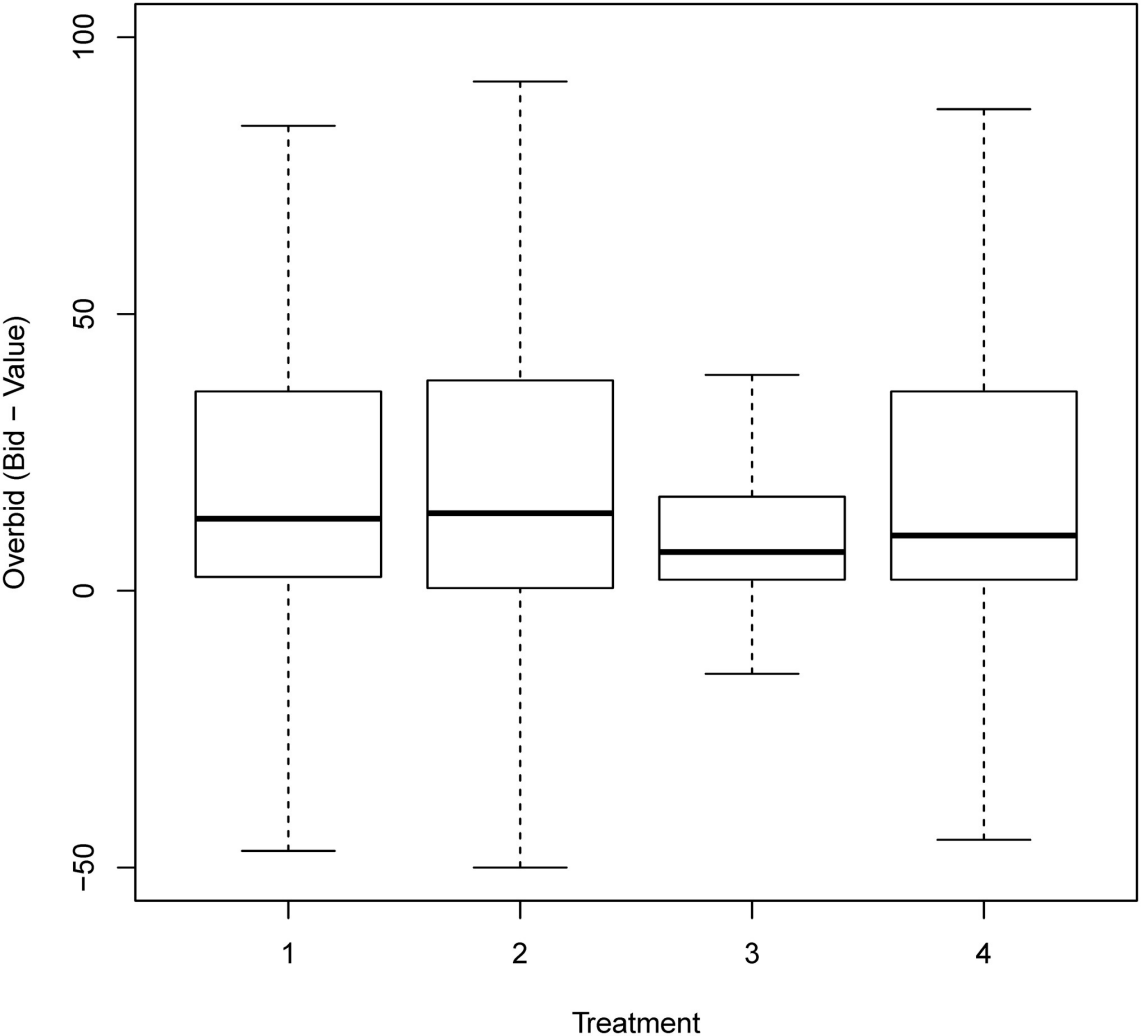
Overbids in all four treatments. Treatment 1: On-the-spot payment; Treatment 2: Lump sum delayed payment; Treatment 3: Delayed endowment; Treatment 4: Prepaid payment.

**Fig 3:**
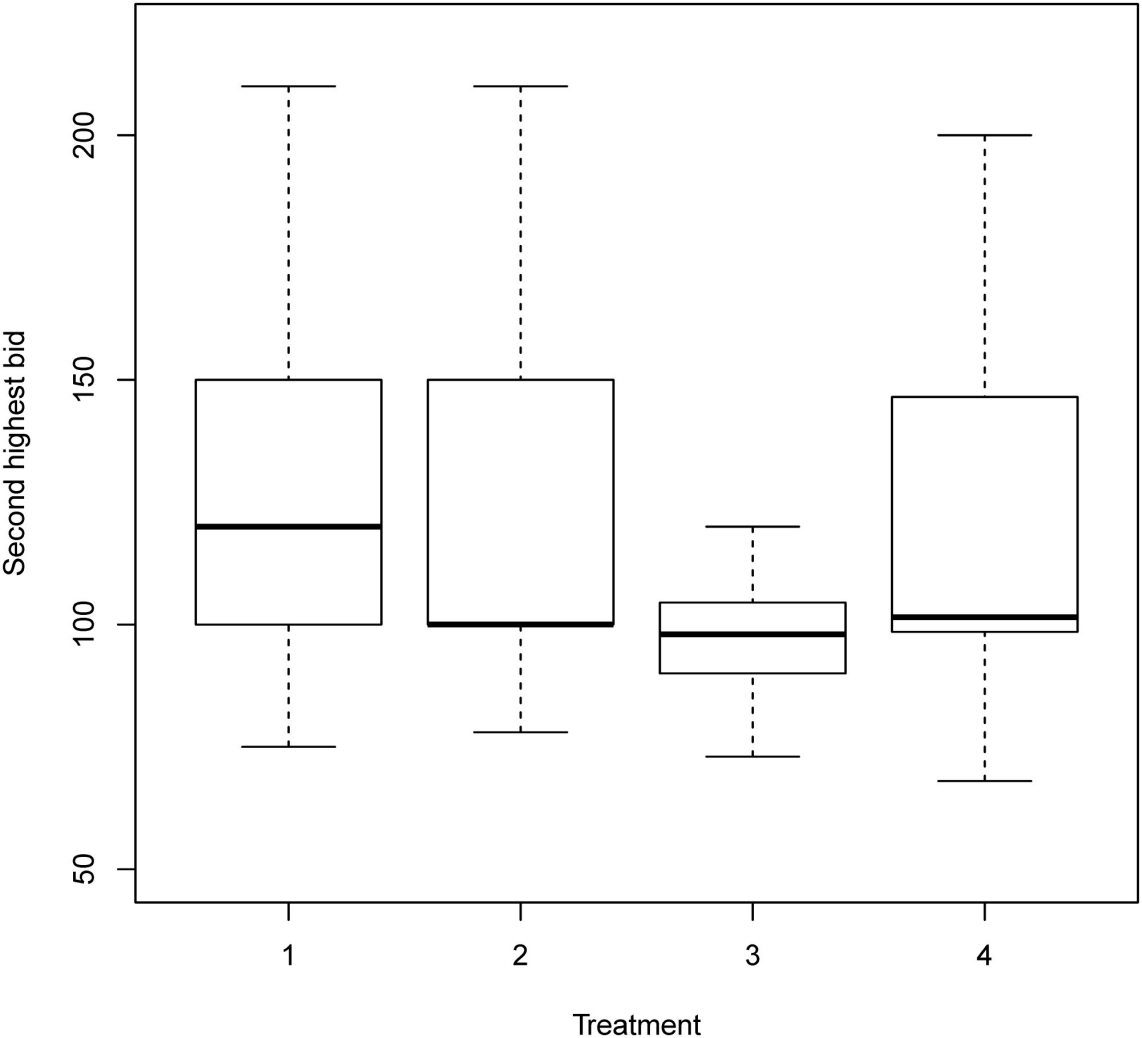
Winning price (the second highest bids) in all four treatments. Treatment 1: On-the-spot payment; Treatment 2: Lump sum delayed payment; Treatment 3: Delayed endowment; Treatment 4: Prepaid payment.

Fig 1 uses the boxplot to describe the distributions of bids. The boxplot presents the interquartile range from 25% to 75% as a rectangle on the chart. The median for the data set is plotted as a solid black line across the rectangle, while the range is plotted with dotted lines extending from the central rectangle up to the maximum and down to the minimum. The average bid of the delayed endowment treatment was significantly lower than that of the “on-the-spot” payment treatment in the last 10 rounds (*p* value <1e-5), while little difference was observed in the first 10 rounds. There was little difference in the average bids between the other two treatments (prepaid and lump sum delayed treatment) and the “on-the-spot” payment treatment. Similar patterns were found for the level of overbid (bid minus value) (Fig 2) and the winning prices (the second highest bids) (Fig 3). On average, subjects in the delayed endowment treatment session overbid by 16ED, while those in the other three treatment sessions overbid by 24ED.

**Fig 4:**
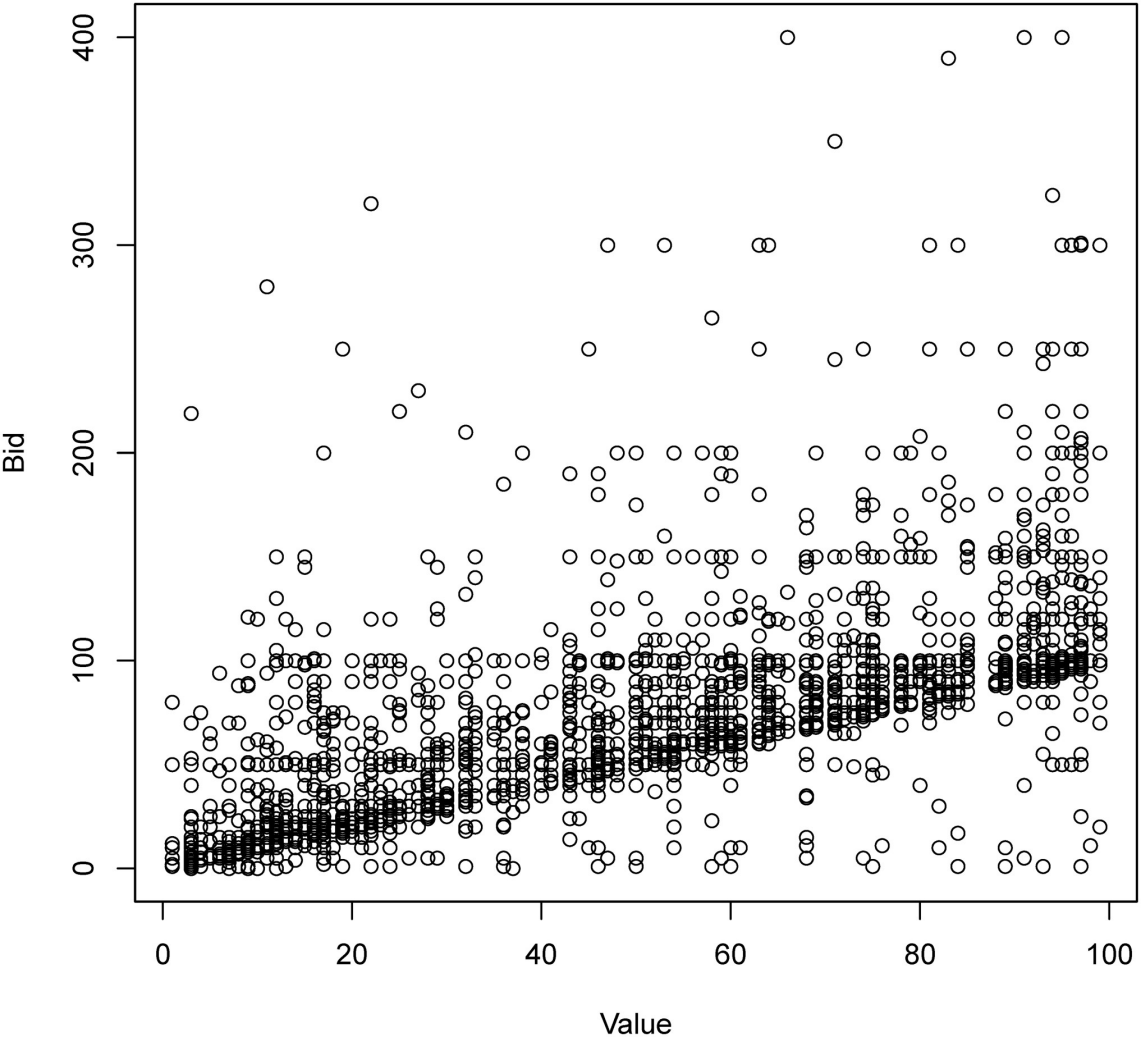
Scatter plot of bids and values.

**Fig 5:**
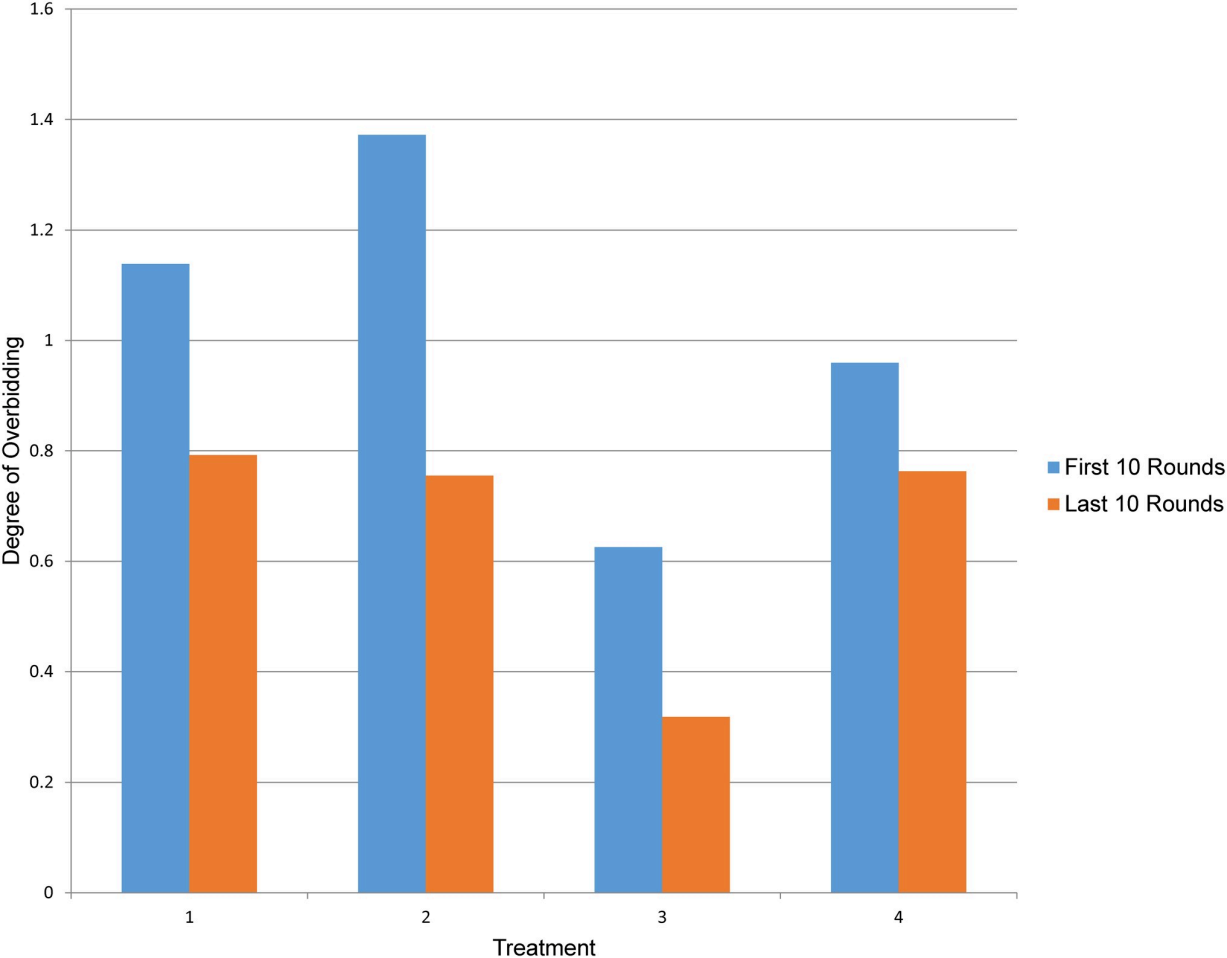
Degree of overbidding in first 10 rounds and last 10 rounds. Treatment 1: On-the-spot payment; Treatment 2: Lump sum delayed payment; Treatment 3: Delayed endowment; Treatment 4: Prepaid payment.

In Fig 4, we plotted subjects’ bids against their corresponding values. There is a clear clustering of observations along the 45 degree line, indicating that many bids are close to the assigned values. At the same time, the majority of the bids are above the 45 degree line, indicating overbidding. The degree of overbidding, defined as [(value–bid)/value x 100%], is shown in Fig 5. In all treatments, the degrees of overbidding were lower in the last 10 rounds than in the first 10 rounds and this difference was particularly significant in the delayed endowment treatment. These results imply that after repeating several rounds of auctions, subjects bid closer to their true values, suggesting the possible existence of bounded rationality and learning, which would be explored using regression analysis in the next section.

### 3.2 Regression analysis

We first employed the linear mixed-effect model to investigate factors that affect bids in the first 10 rounds, the last 10 rounds, and all 20 rounds of auctions, respectively. The treatment effects are modeled by fixed effects, and individual bidder characteristics are modeled by random effects as each individual participated in 20 auction rounds. The explanatory variables in the regressions include the assigned values, treatment dummies, round numbers, risk attitude (CRRA risk parameter), time discount rate, amount of money brought to the experiment (pocket money), age, gender, monthly allowance (= 0 when monthly allowance < PHP 1,999; = 1 when monthly allowance > = 1,999), work status (= 0 if not working; = 1 if either part-time or full-time working), grade point average (GPA), and the amount of accumulated losses up to the previous auction round (lag-accumulated-loss). Note that the monthly allowance in the questionnaire was constructed as a categorical variable (Table 3). We have tested the robustness of the results by constructing dummy variables for all categories and using different cutoff points, which all generated similar results. Therefore, to save degrees of freedom in the regression analysis, we chose to use PHP 1,999 as the cutoff to construct a single dummy variable in the regression analysis for monthly allowance (66 subjects with monthly allowance < 1,999; 54 subjects with monthly allowance > = 1,999).

The estimation results are reported in Table 5. The overall results suggest that the delayed endowment treatment has reduced bids significantly compared to the “on-the-spot” payment treatment especially in the last 10 rounds. Moreover, having a job significantly decreased the bids suggesting that people who worked to earn their money tended to spend their money more carefully, which might be in line with the house money effect. Risk attitudes do not significantly affect bids, suggesting that our results do not appear to provide evidence of the existence of the “joy of winning” effect on overbidding, under which bidders who are more risk averse should be less likely to overbid. The coefficient of “value” was 1.0998 for the first 10 rounds and decreased to 1.0057 for the last 10 rounds. This convergence of bids towards true values in the last 10 rounds implies a possible effect of adaptive learning that reduced overbidding due to bounded rationality in the early rounds of the auctions. The negative coefficients of lag-accumulated-loss suggest that subjects reduce overbidding by learning from previous losses. In addition, bids increased with the amount of money brought to the experiment by subjects in the first 10 rounds.

**Table 5:** Regression results on bids.

	All 20 Rounds		First 10 Rounds		Last 10 Rounds	
	(1)	(2)	(3)	(4)	(5)	(6)
**Intercept**	44.7431 (43.4266)	44.6614 (44.5023)	65.3958 (52.2438)	66.8699 (52.8096)	32.2153 (49.8733)	32.0696 (52.8349)
**Value**	1.0571*** (0.0249)	1.0566*** (0.0249)	1.0998*** (0.0364)	1.0992*** (0.0364)	1.0057*** (0.0312)	1.0050*** (0.0311)
**Lump sum delayed payment**	-2.0343 (6.7674)	-11.6996 (9.9449)	0.2811 (8.1354)	-13.5042 (11.7876)	-4.5148 (7.7417)	-11.1274 (11.7869)
**Delayed endowment**	-13.4755* (6.9199)	-19.6442** (10.0469)	-5.3065 (8.3084)	-15.4788 (11.9040)	-21.9641*** (7.9733)	-25.1436** (11.9338)
**Prepaid**	-1.4774 (6.6720)	-0.6040 (10.5088)	1.8371 (8.0168)	-3.2693 (12.4594)	-4.6031 (7.6333)	0.7981 (12.4325)
**Round#**	0.2283* (0.1329)	0.2403* (0.1330)	0.7663 (0.3597)	0.7869 (0.3597)	-0.0968 (0.3083)	-0.0497 (0.3081)
**Risk attitude**	1.1593 (5.7992)	-0.4808 (6.1649)	-2.9227 (6.9777)	-5.1714 (7.3172)	4.1000 (6.6446)	2.7462 (7.3057)
**Time discount**	-1.9804 (3.0818)	-2.2980 (3.1620)	-0.7123 (3.7030)	-1.3332 (3.7477)	-3.3586 (3.5327)	-3.5610 (3.7496)
**Pocket money**	0.0033 (0.0025)	0.0004 (0.0039)	0.0056* (0.0031)	-0.0003 (0.0046)	0.0013 (0.0029)	0.0013 (0.0045)
**Age**	-1.4801 (2.2417)	-1.1959 (2.3013)	-3.0013 (2.6961)	-2.6336 (2.7301)	0.2214 (2.5631)	-0.0892 (2.7215)
**Female**	-0.2806 (4.9199)	-0.0046 (5.0921)	-1.2039 (5.9124)	-1.5792 (6.0364)	0.4051 (5.6310)	1.4774 (6.0272)
**Monthly allowance**	1.0102 (4.5844)	-4.1322 (9.2297)	4.6825 (5.5120)	-1.3774 (10.9449)	-2.1463 (5.2423)	-7.6049 (10.9219)
**Work status**	-16.6538** (7.3181)	-16.3720** (7.6179)	-19.0114** (8.8057)	-18.0859** (9.0423	-14.5853* (8.3813)	-15.3937* (9.0306)
**GPA**	2.2186 (4.5857)	2.5062 (4.8120)	2.8968 (5.5183)	2.8495 (5.7116)	2.9111 (5.2423)	2.9109 (5.6931)
**Lag-accumulated-loss**	-0.0477*** (0.0156)	-0.0511*** (0.0157)	-0.1767*** (0.0367)	-0.1822*** (0.0333)	-0.0241 (0.0228)	-0.0388 (0.0237)
**Lump sum delayed** **x Pocket money**		0.0062 (0.0059)		0.0116 (0.0070)		0.0012 (0.0070)
**Delayed endowment** **x Pocket money**		0.0089 (0.0077)		0.0168** (0.0092)		0.0014 (0.0092)
**Prepaid** **x Pocket money**		-0.0078 (0.0089)		-0.0030 (0.0117)		-0.0129 (0.0117)
**Lump sum delayed x Monthly allowance**		8.0014 (13.7199)		5.9182 (16.2650)		11.1656 (16.2249)
**Delayed endowment x Monthly allowance**		0.5176 (13.4307)		-3.2119 (15.9227)		4.3875 (15.8817)
**Prepaid x Monthly allowance**		6.2643 (13.2219)		12.1943 (15.6860)		3.6082 (15.6668)
**R**^**2**^	0.4591	0.4568	0.4283	0.4241	0.4019	0.3964

Note: Standard errors are presented in parentheses;

*, **, ***: Statistically significant at the levels of 0.10, 0.05 and 0.01 respectively.

### 3.3 Bounded rationality and learning

We now proceed to examine the possible learning effects by analyzing overbids over auction rounds across different payment treatments. Individual characteristics are controlled by the random effects model. The results are reported in Table 6.

**Table 6:** Regression results on overbids in different treatments.

	On-the-Spot Payment	Lump Sum Delayed Payment	Delayed Endowment	Prepaid Payment
**Intercept**	-147.9238 (94.9257)	115.1398 (107.8659)	33.2282 (94.1374)	131.7060 (110.5959)
**Value**	0.0576 (0.0538)	0.0046 (0.0510)	0.1325*** (0.0398)	0.0458 (0.0507)
**Round#**	-0.1982 (1.1477)	0.5530 (1.4360)	-1.2419 (0.8263)	2.2212 (1.6167)
**Risk attitude**	-8.7466 (15.3756)	28.9954 (29.6026)	32.8690 (30.2821)	-46.7238** (22.0340)
**Time discount**	-1.2650 (5.5221)	13.8768 (26.4273)	40.6001* (23.3000)	59.5878 (45.2545)
**Pocket money**	-0.0018 (0.0042)	0.0077 (0.0062)	0.0089 (0.0076)	-0.0078 (0.0095)
**Age**	7.5681 (4.7898)	-7.4967 (5.5839)	1.6029 (5.0221)	-4.5680 (5.7536)
**Female**	26.8812** (11.6338)	-4.6243 (15.0114)	-7.9207 (11.2651)	-3.0971 (10.3516)
**Monthly allowance**	-6.8188 (9.0607)	-1.4961 (13.3430)	-12.1974 (10.6442)	-6.2364 (10.0595)
**Work status**	-12.1421 (14.4403)	-38.1335** (14.9125)	16.6415 (25.4056)	-10.1313 (20.6920)
**GPA**	3.1897 (9.2087)	18.5925 (14.5640)	-26.3444* (14.5712)	-7.5998 (13.7579)
**Lag-accumulated- loss**	-0.1124*** (0.0331)	-0.0340 (0.0300)	-0.2277*** (0.0000)	-0.0209 (0.0330)
**Round#** **x Risk attitude**	-1.4680* (0.7884)	0.7115 (1.0262)	-1.2980 (1.0063)	4.0567*** (0.0001)
**Round#** **x Time discount**	0.3352 (0.2851)	-0.2690 (0.8997)	-1.0633** (0.7763)	-4.8203** (2.1707)
**Round#** **x GPA**	0.5254 (0.4814)	-0.1779 (0.5718)	0.6093 (0.3892)	-0.9023 (0.6132)
**R**^**2**^	0.8169	0.5201	0.8232	0.7482

Note: Standard errors are presented in parentheses;

*, **, ***: Statistically significant at the levels of 0.10, 0.05 and 0.01 respectively.

In the delayed endowment treatment, overbidding was significantly reduced over rounds. Moreover, in this treatment, overbids were lower for bidders with higher GPA, which is a possible indicator of cognitive ability and learning ability [30, 31]. In the prepaid treatment, where participants had to pay the losses using their own money, as well as in the delayed endowment treatment, overbids were also lower for bidders with higher GPA although the difference is not statistically significant. The interaction term of GPA and number of rounds in the delayed payment treatment is significantly positive, suggesting that individuals with lower GPA learned effectively over rounds. We also found that under the delayed endowment mechanism, overbids were lower for patient bidders compared to impatient bidders. Furthermore, the interaction term of the time discounting rate and number of rounds is negative, meaning that impatient bidders lowered their bid significantly over rounds, which might reflect the learning effect of impatient bidders. Some studies have found that lower cognitive ability is associated with more pronounced impatience [32]. The significant interaction effects of bidding rounds with GPA and time discounting rate in the delayed payment treatment suggest that, bidders with possibly higher degree of bounded rationality were able to learn effectively to reduce overbidding as they gained more experience over auction rounds under this mechanism.

Interestingly, in the prepaid session, an increasing degree of risk aversion decreased overbids significantly. This result is consistent with findings in the previous literature of prepaid mechanism [19]. However this effect is not significant in the delayed payment treatments. The negative coefficients of lag-accumulated-loss in all four payment treatments suggests that subjects learn from losses to reduce overbidding, although this effect is only significant in the “on-the-spot” payment and the delayed endowment treatment possibly due to the small sample size in this study.

### 3.4 Effect of liquidity constraints

In this section, we conducted a close examination of the effect of liquidity constraints, taking into account the pocket money—the amount of money subjects brought to the experiment (we thank Dr. Giovanni Ponti and two anonymous reviewers for the insights of the effect of liquidity constraints on bidding behavior). Subjects’ liquidity constraints might affect their bidding behavior significantly, especially when they have to use their own money to pay for the experimental losses. In our study, each participant was provided with 150PHP, which includes 100PHP participation fee and 50PHP as starting balance for the auctions. During the course of repeated auctions, if a subject’s accumulated profit became lower than -500ED (-50PHP), that is, if the losses became greater than 50PHP, he or she was no longer allowed to bid in subsequent rounds since the subject had exhausted the starting balance of 50PHP. Therefore, there was a loss ceiling of 50PHP for each subject in our experiment. After excluding one outlier who brought 29,000PHP to the experiment, the average amount of money that subjects brought to experiment was 770PHP and the median was 400 PHP. Subjects’ average monthly allowance was around 2,000 PHP and the median was around 1,500 PHP. These amounts were considerably higher than the experiment’s loss ceiling of 50PHP. Furthermore, only 9 out 120 bidders reported pocket money less than the loss ceiling of 50 PHP. Therefore, most subjects did not have liquidity constraints in a strict sense. However, considering the per capita daily consumption of 96 PHP in Philippines (From the National Statistical Coordination Board site (http://www.nscb.gov.ph/secstat/d_income.asp) the annual expenditure per family in the Philippines in 2009 was 176,000PHP. According to National Statistic Office of the Philippines (http://www.philippines.hvu.nl/facts2.htm), the average size of a Filipino family is 5. Therefore, we calculated the per capita daily consumption as 176,000÷365÷5 = 96 PHP.), liquidity constraints became 96PHP + 50PHP = 146 PHP. Under this threshold, there were 32 participants with liquidity constraints, that is, with cash holding of less than 146 PHP on the experiment day. We then conducted the regression analyses separately for participants with or without liquidity constraints. The regression results are presented in Table 7, which shows very different patterns of bidding behavior between these two groups. After 10 auction rounds, bidders with liquidity constraints in all three payment treatment sessions (lump-sum delayed payment, delayed endowment, prepaid payment) bid significantly lower compared to those in the control session (“on-the-spot” payment), whereas for bidders without liquidity constraint, only bidders in the delayed endowment session bid significantly lower than those in the “on-the-spot” payment session.

**Table 7:** Regression on overbids for bidders with/without liquidity constraint.

	Bidders without liquidity constraints (Pocket Money > = 146PHP) (n = 87)		Bidders with liquidity constraints (Pocket Money <146PHP) (n = 32)	
	First 10 rounds	Last 10 rounds	First 10 rounds	Last 10 rounds
**Intercept**	138.4014** (65.8925)	63.7526 (64.6508)	-82.5931 (94.7870)	—65.0902 (99.7029)
**Value**	0.0823* (0.0445)	0.0139 (0.0385)	0.1487** (0.0603)	-0.0198 (0.0489)
**Lump sum delayed payment**	2.6272 (10.5255)	-0.9854 (10.2835)	-17.2941 (14.7559)	-33.4950** (15.8255)
**Delayed endowment**	-4.4058 (11.2019)	-20.0509* (11.0141)	-6.1863 (12.7062)	-31.2452** (13.5900)
**Prepaid treatment**	2.5791 (10.7022)	3.0065 (10.4576)	-1.3001 (11.6957)	-30.0972** (12.3115)
**Round#**	0.8275 (0.4470)	-0.1572 (0.3813)	0.7343 (0.5657)	0.4250 (0.4905)
**Risk attitude**	-1.5423 (8.1988)	4.5875 (8.0114)	9.3848 (17.8013)	-6.2926 (18.6543)
**Time discount**	-2.7236 (4.2577)	-4.2176 (4.1765)	26.3164** (12.5155)	9.6668 (13.1694)
**Pocket money**	0.0066* (0.0038)	-0.0001 (0.0037)	0.0511 (0.1234)	0.1533 (0.1316)
**Age**	-6.0984* (3.3081)	-1.8368 (3.2324)	4.5996 (5.0991)	4.2803 (5.3328)
**Female**	-5.4950 (7.5565)	-4.5596 (7.3802)	-0.5414 (8.8574)	5.8127 (9.3789)
**Monthly allowance**	0.2079 (7.0901)	-5.1944 (0.4561)	8.3501 (8.5084)	1.2600 (8.8940)
**Work status**	-23.8938** (11.2321)	-15.9572 (11.0155)	7.5475 (18.6656)	7.0375 (19.4295)
**GPA**	-0.7831 (7.3989)	5.5888 (7.2280)	-4.7655 (10.0382)	1.3572 (10.4824)
**Lag-accumulated-loss**	-0.2011*** (0.0392)	-0.0304 (0.0271)	-0.1233* (0.0659)	-0.1286** (0.0525)
**R**^**2**^	0.6950	0.6137	0.6844	0.6630

Note: Standard errors are presented in parentheses;

*,**,***: Statistically significant at the levels of 0.10, 0.05 and 0.01 respectively.

These results suggest that cash holdings and liquidity constraints of the subjects could affect bidding behavior significantly. The regression on those without liquidity constraints rules out the possible confounding effect of liquidity constraints and provides further support to the significant effect of delayed endowment mechanism in decreasing overbidding. Interestingly, the effect of value is significantly positive in the first 10 rounds, but not in the last 10 rounds (we thank one anonymous reviewer for suggesting the possible effects of private values on subjects’ perceived probability of winning). This, again, might suggest that overbidding might be caused by bounded rationality. A bidder might mistakenly think that a higher value might lead to higher profit if he won the auction and a higher bid would increase his/her chance of winning. As bidders gained more experience from more auction rounds, their bids converged to the optimal strategy through adaptive learning.

The effect of lag-accumulated-loss is significantly negative on overbidding for both first 10 rounds and last 10 rounds with liquidity constraints, but only significantly negative in the first 10 rounds without liquidity constraints. This result suggests that liquidity constraints and learning may strengthen with each other; i.e., that the incentive to learn is stronger after losing money especially when facing liquidity constraints (we thank two anonymous reviewers for their insights on the interaction effects of liquidity constraints and learning from losses). We speculate that without liquidity constraints, the effect of learning from losing may be diluted by other factors such as bidding war/winner’s curse.

To examine the relationship between the liquidity constraints and probability of overbidding, we conducted a survival analysis by employing the Cox proportional-hazard model, which helps in further disentangling the effects of the payment treatments and the liquidity constraints (we thank an anonymous reviewer for suggesting this method that yields several important results of our study). The Cox proportional-hazards regression is conducted in R using the survival package [33]. In our analysis, the non-survival condition consists of not-overbidding. The conditional probability of overbidding is estimated over the degree of liquidity constraints, which is defined according to the quantile statistics of pocket money with 1 being less than the 5 percentile, and 20 being greater than the 95 percentile (Table 8). The advantage of the Cox proportional hazard model is that, it is a semi-parametric model where the covariates enter the model linearly while the baseline hazard function can take any form [34, 35]. The regression results are presented in Table 9 with hazard ratios computed by exponentiating the parameter estimates.

**Table 8:** Quantile statistics of pocket money.

**Quantile**	5%	10%	15%	20%	25%	30%	35%
**Pocket money (PHP)**	30.95	50.00	80.00	100.00	127.50	150.00	176.90
**Quantile**	40%	45%	50%	55%	60%	65%	70%
**Pocket money (PHP)**	200.00	295.50	425.50	500.00	628.00	800.00	915.00
**Quantile**	75%	80%	85%	90%	95%		
**Pocket money (PHP)**	1025.00	1200.00	1500.00	2145.90	2833.75		

**Table 9:** Cox regression on probability of overbidding conditional on degree of liquidity constraints.

	coef	exp(coef)	se(coef)	P-value
**Value**	0.0025	1.0025	0.0008	0.0024**
**Lump sum delayed payment**	0.3758	1.4561	0.0999	0.0002***
**Delayed endowment**	-0.1723	0.8417	0.0874	0.0487**
**Prepaid**	-0.1488	0.8651	0.1011	0.1519
**Round#**	0.0083	1.0083	0.0040	0.0393
**Risk attitude**	-0.0095	0.9906	0.0725	0.8962
**Time discount**	0.0133	1.0134	0.0387	0.7314
**Pocket money**	0.0081	1.0082	0.0002	<2.2e-16***
**Age**	0.0278	1.0281	0.0256	0.2789
**Female**	0.0530	1.0544	0.0591	0.3693
**Work status**	-0.0010	0.9990	0.0888	0.9911
**GPA**	-0.1508	0.8600	0.0516	0.0035***
**Lump sum delayed payment** * **Pocket Money**	0.0007	1.0007	0.00006	<2.2e-16***
**Delayed endowment** * **Pocket Money**	0.0017	1.0017	0.0001	<2.2e-16***
**Prepaid** * Pocket Money	0.0018	1.0018	0.0001	<2.2e-16***

The p-value of Likelihood ratio test is <2.2e-16

The p-value of Wald test is <2.2e-16

The p-value of Score (log rank) test is <2.2e-16

*, **, ***: Statistically significant at the levels of 0.10, 0.05 and 0.01 respectively.

The Cox proportional-hazards regression shows that, holding the degree of liquidity constraints constant, the probability of overbidding is 17.23% lower (*p* value = 0.0487) in the delayed endowment treatment compared to the control “on-the-spot” payment treatment. The probability of overbidding conditional on the liquidity constraints is also lower in the prepaid treatment compared to the control treatment, although not statistically significant (14.88% lower with *p* value = 0.1519). However, the probability of overbidding conditional on the liquidity constraints in the lump sum delayed payment treatment is significantly higher (37.58% higher with *p* value = 0.0002) than the control group, and this effect was not discovered by the regular linear mixed regression. Under this payment mechanism, liquidity constraints did not matter much on the experiment day, since subjects did not receive any payment or pay the loss and their bidding decisions did not lead to any immediate monetary outcome. While the regular linear mixed regression shows that bidders with liquidity constraints on average bid lower, the Cox proportional hazards regression provides the estimation of the probability of overbidding conditional on liquidity constraints, which differs significantly between different payment treatments. Therefore, the results of Cox regression imply that the role of auction design and that of liquidity constraints may significantly overlap (we thank an anonymous reviewer for important insights in this result). Not surprisingly, subjects who brought more pocket money were more likely to overbid, with every extra PHP increasing the probability of overbidding by 0.81% for the same level of liquidity constraint. The effect of pocket money is stronger in all the other three treatments compared to the control “on-the-spot” payment. Moreover, the subjects with higher GPA are significantly less likely to overbid, with each 1 point increase in GPA (4.0 point scale) lowering the probability of overbidding by 15.08%. Under the assumption that GPA is an indicator of the cognitive ability, this result lends support to the hypothesis that bounded rationality is a driving force of overbidding.

## 4. Concluding remarks

In this study, we designed a “delayed payment” mechanism in economic experiments under which subjects did not receive payment until two weeks after the experiment, and overbidding could cost them their own money on the experiment day. We found that overbidding was reduced significantly under this mechanism compared to the conventional “on-the-spot” payment mechanism. Learning was effective under the delayed endowment payment scheme because it makes overbidding “truly” costly by requiring bidders to pay for their losses with their own money on the experiment day. In our experiments, each auction was repeated for 20 rounds to provide the subjects with sufficient time for learning. Under the adaptive learning theory, rational subjects are expected to learn not to overbid as they gain more experience over rounds when overbidding is costly. Our results are consistent with the adaptive learning theory given that bids converged to the corresponding values over auction rounds and overbidding were reduced by previous losses.

When considering a bidder’s liquidity constraints, we found that both delayed and prepaid payment treatments decreased the degree of overbidding for bidders with liquidity constraint. However, in the situation without liquidity constraints, overbidding was reduced by the delayed payment of endowment but not by the prepaid mechanism. It is possible that subjects who received endowment two weeks before the experiment were more willing and prepared to pay than the ones who did not receive the endowment yet. Furthermore, when controlling the degree of liquidity constraints, we found that subjects with higher GPAs were less likely to overbid and the delayed endowment mechanism significantly reduced overbidding compared to other payment mechanisms. Therefore, overbidding seems to depend significantly on bidders’ cash holding, and the magnitude of the payment treatment effects depends crucially on liquidity constraints. A major limitation of this study is the small sample size, which may lead to the insignificant effects of some important explanatory variables. Further research should be done with a larger number of subjects. In summary, the results of our study lend support to the hypothesis that overbidding in SPAs might be caused by bounded rationality and could be reduced by adaptive learning. A payment mechanism that makes overbidding “truly” costly and also provides bidders enough learning experience can reduce overbidding.
